# On the melting point depression, coalescence, and chemical ordering of bimetallic nanoparticles: the miscible Ni–Pt system

**DOI:** 10.1039/d2na00418f

**Published:** 2022-10-20

**Authors:** Evropi Toulkeridou, Joseph Kioseoglou, Panagiotis Grammatikopoulos

**Affiliations:** Okinawa Institute of Science and Technology Graduate University 1919-1 Tancha, Onna-Son Okinawa 904-0495 Japan panagiotis.g@gtiit.edu.cn; Department of Physics, Aristotle University of Thessaloniki GR-54124 Thessaloniki Greece; Department of Materials Sciences and Engineering, Guangdong Technion – Israel Institute of Technology Shantou Guangdong 515063 China; Guangdong Provincial Key Laboratory of Materials and Technologies for Energy Conversion, Guangdong Technion – Israel Institute of Technology Shantou Guangdong 515063 China

## Abstract

Among the properties that distinguish nanoparticles (NPs) from their bulk counterparts is their lower melting points. It is also common knowledge that relatively low melting points enhance the coalescence of (usually) nascent nanoclusters toward larger NPs. Finally, it is well established that the chemical ordering of bi- (or multi-) metallic NPs can have a profound effect on their physical and chemical properties, dictating their potential applications. With these three considerations in mind, we investigated the coalescence mechanisms for Ni and Pt NPs of various configurations using classical molecular dynamics (MD) computer simulations. Benchmarking the coalescence process, we identified a steeper melting point depression for Pt than for Ni, which indicates a reversal in the order of melting for same-size NPs of the two elements. This reversal, also evident in the nano-phase diagram thermodynamically constructed using the regular solution model, may be useful for utilising NP coalescence as a means to design and engineer non-equilibrium NPs *via* gas-phase synthesis. Indeed, our MD simulations revealed different coalescence mechanisms at play depending on the conditions, leading to segregated chemical orderings such as quasi-Janus core-satellite, or core–(partial) shell NPs, despite the expected theoretical tendency for elemental mixing.

## Abstract 2

We elucidate the coalescence mechanisms of miscible binary nanoparticles based on their nanophase diagrams to inspire their tailored gas-phase synthesis.

## Graphical abstract

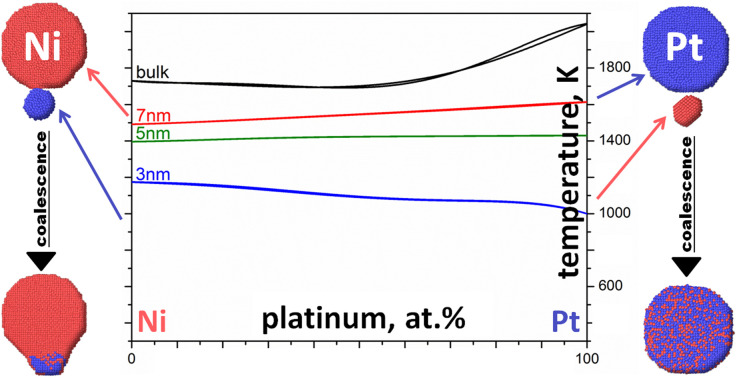

## Introduction

According to classical thermodynamics, melting is determined by the Gibbs free energy difference between the liquid and solid states.^1^ At the nanoscale, a size and a shape parameter need also be introduced to express the melting point of nanoparticles (NPs) as a function (a fraction, in principle) of that of the bulk materials. Their lower melting points are among the properties that distinguish NPs from their bulk counterparts.^2,3^ Indeed, the graph showing the melting point depression of Au as a function of NP diameter regularly appears at introductory nanotechnology classes^4^ as a typical example of a physical property within the scalable regime of the nanoworld.^5^

One of the most characteristic consequences of the melting point depression of NPs is that it enhances their coalescence,^6^ an exemplar process where bulk and nano matter's behaviour differentiate not only quantitatively but also qualitatively.^7^ Indeed, for macroscopic objects (*e.g.*, two solid metallic balls) to fuse together, high sintering temperatures are necessary, whereas solid metallic NPs can fully coalesce even at room temperature due to their dangling bonds and assisted by their lower melting points.^6^

This fundamental physical process became technologically relevant with the advent of various techniques for the gas-phase synthesis of NPs.^8–10^ Unlike their counterparts fabricated by chemical synthesis, NPs grown from the gas phase assume morphologies governed solely by basic physical processes. Coalescence between small (usually nascent) clusters toward bigger NPs in-flight^11–13^ is among the most common growth mechanisms, often dictating NP size and structure. Moreover, due to their high surface area NPs deposited on a substrate are inherently unstable and often tend to coalesce into larger NPs (a process known as Smoluchowski ripening^14^). This can lead to a reduction of the total surface area of, *e.g.*, a catalytically active phase, rendering NP coalescence one of the main culprits for catalyst deactivation. As a result, a great number of studies, both experimental and theoretical, focused on NP coalescence over the past decades.^15–22^ Among their common findings was the effect NP coalescence can have on the chemical ordering of nanoalloys,^23–26^ which, in turn, can largely determine their physical and chemical properties, thus dictating their potential applications.^27–30^

With these considerations in mind, we investigated the coalescence mechanisms for Ni and Pt NPs using classical MD computer simulations. The Ni–Pt system, which can be employed in a wide range of industrial applications to enhance catalytic activities,^31–33^ is strongly miscible, as indicated by its bulk phase diagram,^34^ and this study complements our previous investigation of the Ag–Cu system,^35^ which is practically immiscible.^5^ Our goal was to pinpoint potential differences and/or similarities between such diametrically opposed systems (as per their mixing/demixing behaviour), which would highlight important aspects of the coalescence mechanisms.

Benchmarking the coalescence process, we identified a reversal in the order of melting for same-size NPs of the two elements. This reversal was also evident in the nano-phase diagram constructed nano-thermodynamically using the methodology by Guisbiers *et al.*^36–38^ Nano-thermodynamics^39,40^ is a fundamental approach that allows constructing nano-phase diagrams, considering that the limited number of atoms present in nanosystems do not guarantee the applicability of classical thermodynamics. Based on this finding, we utilised different conditions for NP coalescence and designed core–shell or (quasi-)Janus NPs, which may provide insights for the experimental fabrication of metastable miscible-yet-demixed NPs to nanoscientists and nanotechnologists working in the field of gas-phase synthesis of NPs.

## Computational methods

Molecular dynamics (MD) calculations were performed with LAMMPS,^41^ utilising an embedded-atom method (EAM) inter-atomic potential.^42,43^ All the bulk Ni–Pt compounds and related NPs were formed with the use of the Density Functional Theory (DFT) based structures from the Materials Project^44^ and the NanomaterialsCad tool.^45^ For the MD simulations a default timestep size of 0.001 ps was used, and at least 25 000 000 MD steps were performed for the smallest NPs (3 nm in diameter), corresponding to a minimum 25 ns simulation time. Visualisation was performed using OVITO;^46^ Common Neighbour Analysis (CNA)^47^ using adaptive CNA (a-CNA)^48^ was performed within OVITO, which determines optimal cut-off radii for nearest-neighbours automatically for each individual atom in multiphase systems.

We calculated the melting temperatures of single-element Ni and Pt NPs in the size regime of interest by plotting their caloric curves using MD. Near-spherical fcc NPs were assumed, initially cut from bulk structures and relaxed statically, containing 1288 and 1435 (3 nm), 5971 and 6650 (5 nm), and 16 287 and 18 140 (7 nm) Ni and Pt atoms, respectively. We equilibrated each NP at discrete temperature intervals (from 400 K up to 2000 K with a step of 100 K, implemented in the NVT ensemble) and plotted its potential energy as a function of temperature. For each distinct simulation, sufficient time was provided until the potential energy was stabilised and fluctuations subsided for at least 5 ns; the average potential energy per atom of the last 1 ns was used as a data point for each temperature of the caloric curve. A sharp increase in this plot's slope (stemming from a sudden release of heat of fusion) was a clear fingerprint of the onset of melting; thus, the meting point of each NP was determined with an accuracy of 100 K. Subsequently, nine more temperatures were examined, this time with a temperature step of 10 K, between the temperatures where the sharp slope increase was identified. Consequently, the achieved maximum accuracy of the melting point calculations is equal to 10 K.

The coalescence between (i) equally sized Ni and Pt NPs of various diameters (3, 5, and 7 nm) as well as between (ii) large-diameter Ni (3, 5, and 7 nm) and small-diameter Pt (1.5, 1.9, and 2.5 nm) NPs and (iii) small-diameter Ni (2.5 nm) and large-diameter Pt (7 nm) NPs was investigated at various temperatures below and in-between their estimated melting points. First, the NPs were relaxed individually at various temperatures using the canonical (NVT) ensemble. Next, they were inserted in the same simulation box at a distance just short of the cut-off radius of the inter-atomic potential (6.198 Å), and their coalescence was investigated using the microcanonical (NVE) ensemble to allow for their heating due to surface energy annihilation, according to the scheme detailed in ref. ^7^.

## Results and discussion

### 1. Melting point depression

#### a. MD-simulated caloric curves

Before performing any coalescence studies, it is useful to know the melting temperatures of the initial NPs for the sizes of interest. As shown previously through the extended Cluster Heating Model (e-CHM) for same-element NPs,^6^ sintering behaviour is heavily influenced by the melting points and the proximity of the current temperature to them. When the current temperature approaches *T*_melt_, the clusters can fully fuse together very fast (*i.e.*, within the time limits of atomistic simulations, typically in the order of tens or hundreds of ns^7^). When the current temperature is well below the *T*_melt_, coalescence between the clusters slows down significantly and is, in practical terms, often limited.

**Fig. 1:**
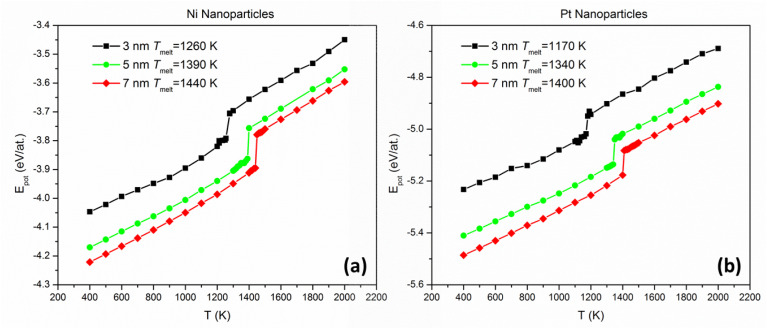
Dependence of potential energy on temperature for single (a) Ni and (b) Pt NPs, 3, 5, and 7 nm in diameter. Sudden increases in slope signify the onset of melting.

We plotted the caloric curves of Ni and Pt NPs 3, 5, and 7 nm in diameter. From the caloric curves (Fig. 1) we observe that, in the size range investigated, Pt NPs have consistently lower melting points than Ni NPs, as noted in the figure. However, it can also be seen that the difference in melting temperatures (*T*^Ni^_melt_ − *T*^Pt^_melt_) decreases with increasing size. This indicates that a melting point inversion threshold-size should exist, also considering that bulk Pt has a higher melting point than bulk Ni (1837 *vs.* 1513 K, according to previous studies utilising the same inter-atomic potential as the current study^49,50^).

#### b. Construction of nanophase diagram

To investigate this point further, we utilised the regular solution model methodology by Guisbiers *et al.*^51^ for the construction of an elementary nanophase diagram of the system for the sizes considered. Its main premise is that the melting point is not only size dependent but also shape dependent; the shape itself being determined by the crystallographic orientation of the facets.^52^ The method has been shown to work well for miscible systems^36–38^ such as the one described here. In fact, Guisbiers *et al.* also constructed nanophase diagrams for the Ni–Pt system as well^53^ but using a slightly different version of the bulk phase diagram than in the present work^34^ and for different NP sizes. The method also has limitations in accuracy, related to certain implicit assumptions. For example, the regular solution model implies perfect shapes, with no deviations from ideality. Clearly this is not the case in either MD simulated NPs (*e.g.*, truncated octahedral NPs of small sizes contain small (110)-type facets, whereas platonic truncated octahedra do not) or experimentally fabricated NPs. Further, the method becomes less precise below 4 nm in diameter, due to weak size dependences in the lattice constant and surface energies. Nevertheless, it suffices for our purpose here, which is to qualitatively explore the possibility of melting point order inversion between Ni and Pt NPs.

In the regular solution model,^39^ the solidus–liquidus curves are given by:1
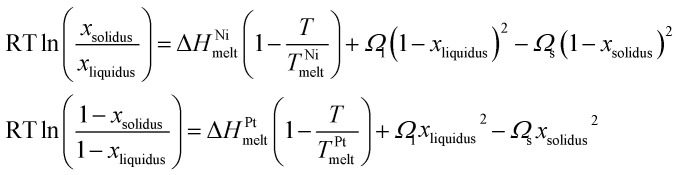
where *R* is the characteristic gas constant, *x*_solidus_ (*x*_liquidus_) is the composition of the solid (liquid) phase at given temperature *T*, *T*^Ni^_melt_ and *T*^Pt^_melt_ are the size-dependent melting temperature of Ni and Pt, respectively, Δ*H*^Ni^_melt_ and Δ*H*^Pt^_melt_ are the size-dependent melting enthalpy of Ni and Pt, respectively, and *Ω*_l_ and *Ω*_s_ are the size-dependent interaction parameters/energy in the liquid and solid phases, respectively.^36,54^ For any specific *T* value, *x*_solidus_ and *x*_liquidus_ are both unique in the bulk phase diagram and can be calculated when the other quantities are known.

For specific values of *T* and for *x*_solidus_ and *x*_liquidus_ specified from the corresponding bulk phase diagram, *Ω*_l,∞_ and *Ω*_s,∞_ can be determined by using the eqn (1). Since *Ω*_l,∞_ and *Ω*_s,∞_ are only weak functions of composition, as a first-order approximation they can be determined at the corresponding *x*_solidus_ and *x*_liquidus_ at *T* ≅ (*T*^Ni^_melt_ + *T*^Pt^_melt_)/2 of their bulk phase diagram.^54^

For the transition from the bulk to the nanoscale phase diagrams, all the size-dependent parameters need to be re-evaluated. To do so, a linear function of 1/*D*, where *D* is the length edge of a polyhedral NP, is used:2
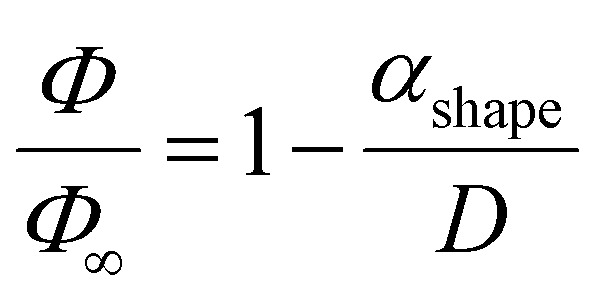
where *Φ* and *Φ*_∞_ are the nanoscale and bulk property parameters, respectively (*i.e.*, *T*^Ni^_melt_ and *T*^Pt^_melt_, Δ*H*^Ni^_melt_ and Δ*H*^Pt^_melt_, *Ω*^Ni^_l_ and *Ω*^Pt^_s_).^36^ The shape-dependent parameter *α*_shape_, which quantifies the size effect on the material property, is defined as:3
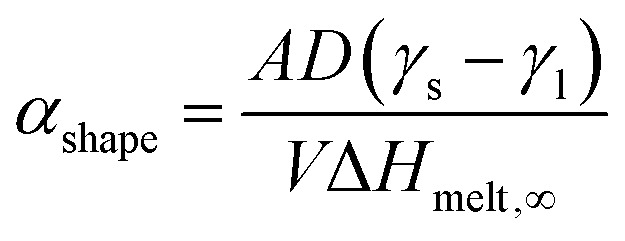
where *A*/*V* is the surface area-to-volume ratio, Δ*H*_melt,∞_ is the bulk melting enthalpy, and *γ*_s(l)_ is the surface energy in the solid (liquid) state.^51^ The ratio between the number of surface atoms and the total number of atoms can be generalised as:4
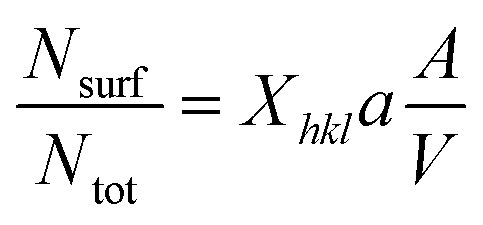
where *X*_*hkl*_ is a numerical constant which only depends on the crystal orientation, and *a* is the bulk lattice parameter.^47^

Combining eqn (2)–(4) we get:^36^5
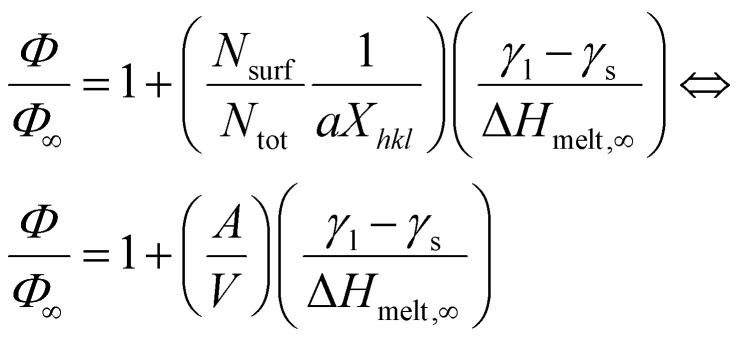


For every material and for different sizes we calculate:^51^
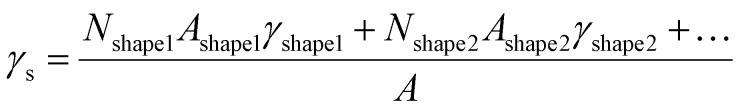
and derive, *A*, *V* geometrically, using surface area and volume equations that correspond to the specific shape of the NP. As a result, all quantities in eqn (5) are known, and *Φ* can be, thus, calculated.

**Fig. 2:**
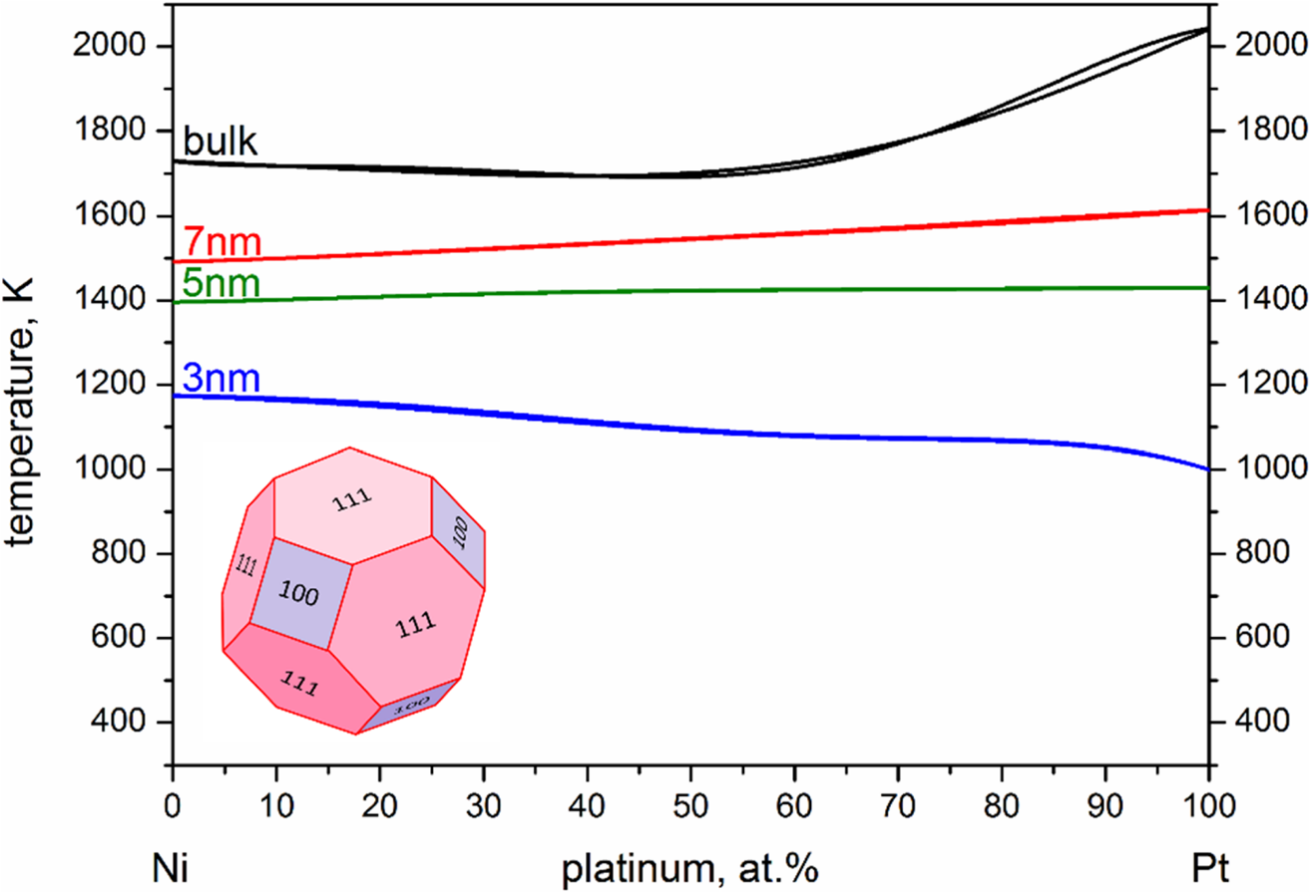
Calculated nanophase diagram for the Ni–Pt binary system. The black, red, green, and blue curves indicate the melting temperatures of the alloy for the bulk, and truncated-octahedral NPs 3, 5, and 7 nm in body diagonal, respectively. A clear melting point order inversion is observed between 3 and 7 nm NPs, whereas 5 nm Ni and Pt NPs share similar melting temperatures.

For our calculations we entered values corresponding to truncated octahedral shapes, based on the expected structures for fcc NPs of both elements (calculations based on the regular solution model^51^ indicated the dodecahedron shape as slightly more stable for Ni; however, we have consistently found the truncated octahedron as the equilibrium shape for similar sizes in previous MD studies^55^). All the values taken from literature of materials properties^56,57^ necessary for the calculation of the nanophase diagrams are tabulated in Table 1; a similar table containing identical values for Ni and Pt (among other elements) also exists in ref. 49, since the authors used the same literature sources. Table 2 contains all the *Φ* values for NPs 3, 5, and 7 nm in body diagonal. Based on these values, we constructed the nanophase diagram of Fig. 2.

**Table 1:** Material property values from the literature used for the calculations. We opted for a limited number of sources for consistency

Materials properties	Ni	Pt
Crystal structure	fcc	fcc
*T*_melt,∞_ (K)^57^	1728.3	2041.5
Δ*H*_melt,∞_ (J mol^−1^)^57^	17 480	22 170
*γ*_s,111_ (J m^−2^)^56^	2.011	2.299
*γ*_s,100_ (J m^−2^)^56^	2.426	2.734
*γ*_s,110_ (J m^−2^)^56^	2.368	2.819
*γ*_l_ (J m^−2^)^57^	1.725	1.866

**Table 2:** Calculated *Φ* values for truncated-octahedral NPs 3, 5, and 7 nm in body diagonal

	*T* (K)		Δ*H* (J mol^−1^)		*Ω*_s_ (J mol^−1^)		*Ω*_l_ (J mol^−1^)		*Φ*/*Φ*_∞_	
	Ni	Pt	Ni	Pt	Ni	Pt	Ni	Pt	Ni	Pt
Bulk	1728	2041	17 480	22 170	153 477		162 946		—	
3 nm	1175.04	1000.3	11 886.4	10 863.3	104 364.36	75 203.7	110 803.3	79 843.5	0.68	0.49
5 nm	1396.224	1429.05	14 123.84	15 519	124 009.4	107 433.9	131 660.4	114 062.2	0.808	0.7
7 nm	1491.26	1614.82	15 085	17 536.47	132 450.6	121 400.3	140 622.4	128 890.3	0.863	0.791

#### c. Comparison between simulated and thermodynamics results

**Fig. 3:**
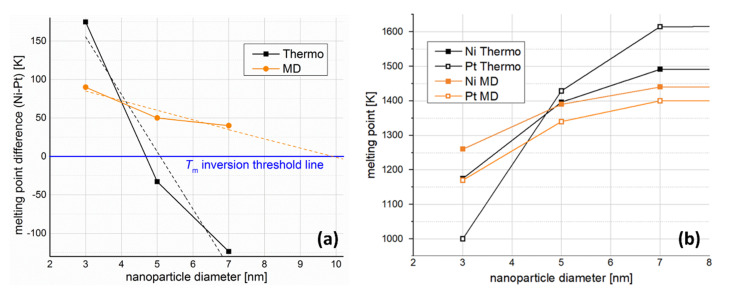
(a) Melting point difference (*T*^Ni^_melt_ − *T*^Pt^_melt_), and (b) melting temperatures as a function of NP size, calculated both by nano-thermodynamics and MD simulations. Both methods indicate a melting point order inversion, even though they point toward different numerical values for its threshold size.

It is evident from the nanophase diagram that it reproduces the aforementioned decrease in melting point difference (*T*^Ni^_melt_ − *T*^Pt^_melt_) with increasing size, as explicitly depicted in Fig. 3a (orange and black lines for MD and nano-thermodynamics results, respectively). However, there is a striking quantitative disagreement between this nano-thermodynamics treatment and the MD-calculated melting points: namely, the melting point inversion threshold-size lying within the investigated size range. This is more clearly shown in Fig. 3b, where it is evident that the simulated melting point lines (in orange) do not cross, whereas the thermodynamically calculated ones (in black) do.

Linear extrapolation of the MD plot in Fig. 3a (orange dashed line) indicates that NPs of the two elements would share the same melting temperature at size of 9.8 nm. Of course, one should note that the curve is probably not linear, which would offset the inversion point toward significantly larger sizes in reality. No matter where this point lies, however, beyond that point Pt NPs should have higher melting temperatures than Ni NPs of the same size. In a similar fashion, linear interpolation of our nano-thermodynamics results (black dashed line) indicates that Ni and Pt NPs 5.1 nm in diameter share the same melting point. Once again, linearity is a rather crude approximation, as the melting point inversion appears to happen earlier, at around 4.6 nm in diameter.

Despite the quantitative difference, which was expected due to the different implicit approximations between the two methods, both studies indicate a steeper melting point depression for Pt. This means that a melting point order inversion threshold-size exists, considering the higher melting temperature of bulk Pt compared with that of Ni, with the corresponding changes in behaviour of the coalescing system.

### 2. MD coalescence studies

**Fig. 4:**
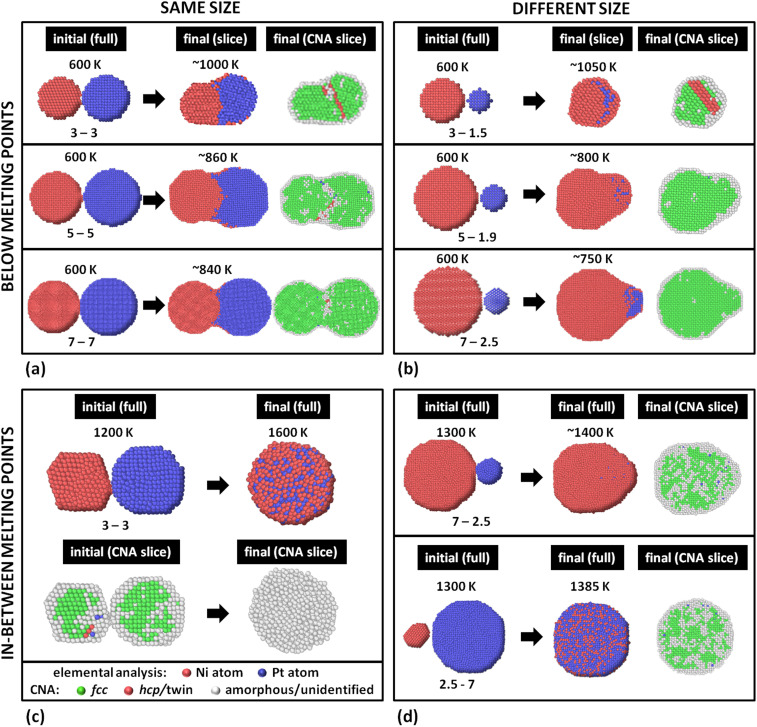
Initial and final configurations of four groups of MD simulations of a single Ni and a single Pt NP (of various sizes) coalescing at different initial temperatures. Each NP size (in nm) is indicated in the figure, as well as the initial and final temperatures (the temperatures rose due to surface energy annihilation in the NVE ensemble). (a) Group 1: below *T*_melt_'s, same-size NPs. (b) Group 2: below *T*_melt_'s, different-size NPs. (c) Group 3: in-between *T*_melt_'s, same-size NPs. (d) Group 4: in-between *T*_melt_'s, different-size NPs. The NPs in each instance are shown either fully or as slices through their equatorial planes; also slices depicting the crystallographic structures of the NPs are shown (CNA slices). In elemental analysis, Ni and Pt atoms are depicted red and blue, respectively. In CNA analysis, atoms arranged in fcc formations are depicted green, whereas twin boundaries are indicated by red atoms (twin atoms are identified as arranged in hcp orientations by OVITO). Surface atoms or atoms in amorphous formations are shown white. Individual atoms are momentarily identified by OVITO to assume bcc coordination; such atoms are depicted blue. Clearly, a variety of non-equilibrium structures can be generated using different coalescence conditions.

We performed coalescence studies divided in four groups. Knowing that the very end of these simulations, if prolonged enough, would contain mixed configurations in regular-shaped NPs, we stopped the runs at relatively early stages of the coalescence process (between ∼60 and 150 ns). Previous experience has shown that these configurations often resemble NPs grown by some gas-phase synthesis method^8,55,58^ better than equilibrium ones, due to the fast quenching experimental NPs experience while entering the deposition chamber.^35^ The initial and final configurations of our MD simulations of all four groups are summarised in Fig. 4.

#### a. Group 1: below *T*_melt_'s, same-size NPs (Fig. 4a)

The sizes studied are those for which we calculated the melting points. NVE ensembles were used at temperatures well below the melting points; both NPs were thermalised at 600 K. This corresponds to experimental temperatures (typically around room temperature) plus a few hundred degrees for speeding up the processes without tampering with phase transitions or significantly modifying the kinetics qualitatively. After agglomeration the temperature rose due to surface energy annihilation and stabilised at ∼1000, 860, and 840 K for 3, 5, and 7 nm in diameter, respectively. The stabilised temperatures are analogous to the degree of coalescence for each system, which was higher for small NPs and decreased with increasing NP sizes. In principle, temperature increase due to high degree of coalescence promotes coalescence further, which in turn evokes further temperature increase in a loop of positive feedback, as elaborated in ref. 6.

The NPs retained their crystallinity during sintering (Common Neighbour Analysis – CNA – slices). This is possible since this miscible system has a basic bulk phase diagram, which means no melting point drops are expected for intermediate alloy Ni–Pt compositions. This is in contrast to the case of eutectic systems (*e.g.*, Ag–Cu^35^), where the melting point of the alloy drops near the eutectic composition, and, as a result, the alloy NPs melt at lower temperatures than the *T*_melt_'s of their constituents. Here, the final configuration retained its fcc structure, with potential formation of a twin boundary near (but not exactly at) the original interface of the two adjacent NPs (case 3–3 nm). This relocation of the boundary within the body of one or the other NP has been explained in detail before,^18^ and is a result of the interfacial atoms temporarily loosening their bonds with their surrounding atoms.^6^

Interestingly, while in this group of simulations Pt NPs always have lower melting points than Ni NPs, Ni atoms show a tendency to diffuse more, mostly on the surface. This stems from two facts. First, the current temperature is well below either melting point, making the Δ*T*_melt_ irrelevant. Further, Pt atoms have a higher cohesive energy,^59^ meaning that Pt clusters behave as single cohesive objects, whereas Ni clusters resemble loosely connected collections of atoms which are easily detached to drift away. This is analogous to the behaviour of atoms in our previous study of the eutectic Ag–Cu system,^35^ with Cu and Ag clusters respectively corresponding to Pt and Ni clusters of the present study. According to Table 1, Ni has a lower surface energy in all crystallographic directions (100), (110), and (111), and in liquid, too; this explains the tendency for quasi-Janus structures with a Ni surface monolayer.

#### b. Group 2: below *T*_melt_'s, different-size NPs (Fig. 4b)

For this group of initial configurations we produced the following initial configurations:

(1) Ni NP: 3 nm – Pt NP: 1.5 nm; at 600 K, stabilised at 1050 K.

(2) Ni NP: 5 nm – Pt NP: 1.9 nm; at 600 K, stabilised at 800 K.

(3) Ni NP: 7 nm – Pt NP: 2.5 nm; at 600 K, stabilised at 750 K.

For the duration of our simulation runs (145, 110, 85 ns, respectively), we observe the same tendency for quasi-Janus formation as with the previous group, with Ni atoms diffusing on the surface to form the external layer of the mixed NPs. Due to the size difference, the coalesced system can be better described as core-satellite. In the former case, the small Pt NP melts momentarily and wets the Ni NP, before its atoms reposition themselves within the crystalline Ni matrix. CNA indicates twinning near the interface between the Ni NP and the Pt precipitate inside it; however, the twinning does not coincide with the interface or any of the two phases, indicating that the Pt atoms aligned epitaxially with the larger Ni matrix, the stacking fault not-withstanding. The CNA of the latter two cases (5–1.9 and 7–2.5 nm) indicates that the Pt NP flattens slowly (due to the relatively low temperature), also covering the surface of the Ni NP epitaxially. No boundary is observed in the slice images.

As a result, we shifted our focus towards systems where the current temperature was above the melting point of the one NP but below that of the other, to investigate if it would be possible to wet the surface of the latter with the atoms of the former.

#### c. Group 3: in-between *T*_melt_'s, same-size NPs (Fig. 4c)

We simulated NPs 3–3 nm in diameter at 1200 K (melting points: 1260 and 1170 K for Ni and Pt NPs, respectively). Utilisation of the micro-canonical NVE ensemble, however, allowed the temperature to rise to 1600 K; therefore, both NPs melted during sintering due to their initial Δ*T*_melt_ being rather small, and their atoms mixed. As a result, the end product was a random-solution liquid NP. Fig. 4c serves as an exemplary visual confirmation of e-CHM's premise, namely, the importance of proximity of the current temperature to the melting points of the NPs.

#### d. Group 4: in-between *T*_melt_'s, different-size NPs (Fig. 4d)

We investigated at 1300 K a configuration containing a large Ni and a small Pt NP (7 and 2.5 nm in diameter, *T*^Ni^_melt_ = 1440 K and *T*^Pt^_melt_ < 1170 K) and *vice versa* (2.5 and 7 nm in diameter, *T*^Ni^_melt_ < 1260 K and *T*^Pt^_melt_ = 1400 K). In the former case, the temperature increased to 1400 K; as a result, the small Pt NP was flattened and wetted the Ni NP, which remained solid and mostly retained its crystallinity. Eventually, the Pt satellite recrystallised epitaxially with the Ni core. Overall, the process was slow due to Pt's high cohesion, and never reached completion within the timeframe of our simulation. A core-satellite configuration formed at the end of the run with quasi-Janus characteristics, as Ni atoms formed a monolayer on top of the Pt satellite.

In the latter case, the temperature remained at similar levels (namely, 1385 K) but the coalescence mechanism was different: the mobile atoms of the molten Ni NP diffused on the surface of the large Pt NP non-hermetically encapsulating it with a surface sub-monolayer in a core@partial-shell configuration.

### 3. Regarding chemical ordering

Our MD study was an *in silico* exercise to explore the configurational variation one can generate by tuning different parameters affecting the coalescence mechanism of a fully miscible bimetallic system. To this end, we investigated the melting point inversion as a potential cause for modification of the chemical ordering of the coalesced system. Since no Δ*T*_melt_ between equal-size NPs in the diameter range we studied was pronounced enough to compensate for the heating due to surface annihilation upon coalescence, we also used the same approach for different-size NPs.

Group 1 results indicate that when the current temperature is way below *T*_melt_ the coalescence mechanism is dominated by the cohesive energies of the NPs (and their differences). Chemical ordering depends heavily on surface energies (and their differences). As our miscible system fulfils the Hume-Rothery criteria regarding crystal structure, atomic radii, valence, and electronegativity, no demixing tendencies should be expected.^60^ However, the significantly lower surface energy of Ni, assisted by its low cohesion, allowed Ni atoms to surface-diffuse and form a (sub)-monolayer shell. This, in principle, constitutes segregation rule #2, as defined by Guisbiers *et al.*^38^ However, it should be noted that according to that study rule #2 should become relevant if the difference between the bulk melting temperatures of the two elements is no larger than 10% of bulk *T*^Pt^_melt_. In this case, though, Δ*T*_melt_ = 313 K, *i.e.*, ∼15% of bulk *T*^Pt^_melt_. Therefore, either the importance of the bulk *T*_melt_ was overrated, or, possibly, the *T*_melt_ of the actual NPs should be considered instead.

Despite their superficial similarity, there is a difference between cases 7–2.5 of Groups 2 and 4. In the former, the Pt NP retains its crystallinity throughout the whole process, whereas in the latter it temporarily melts before the energy dissipates in the system and it recrystallises. This emphasises the role of kinetics, as two phenomena happen simultaneously: wetting of Pt on Ni, and surface diffusion of Ni atoms. Their relative rates may define at any given time the oblongness of the coalesced NP or the presence/absence of a mixed phase in the Pt-rich region.

Finally, it is noteworthy that, except for the random-solution formation of Group 3 where both NPs melted, no other configuration (quasi-Janus, core-satellite, or core–shell) corresponds to equilibrium chemical ordering. Instead, all generated structures are equivalent to those of the immiscible Ag–Cu system. This is in concord with the findings of Guisbiers *et al.*^36^ for the Cu–Ni nanoalloy, another ideal substitutional solid solution system, which can form mixed or Janus NPs depending on the synthesis temperature. In conclusion, it appears that the method is insensitive to the miscibility of the system, which corresponds well to experimentally fabricated NPs from the gas phase in highly non-equilibrium conditions.

## Conclusions

We investigated by classical MD the coalescence mechanisms of Ni and Pt NPs, which, in the bulk, constitute a miscible binary system. Utilising both caloric curves by MD and nano-thermodynamics, we identified an inversion in the order of the melting points of the two different-element NPs with increasing size, which meant that, in principle, we could have either type melt and wet the other, if the current temperature was chosen in-between these melting points. We identified different coalescence mechanisms at play depending on the conditions, leading to different configurations. Segregated chemical orderings were thus produced, such as quasi-Janus core-satellite, or core–(partial) shell NPs, regardless of the expected theoretical tendency for elemental mixing. In fact, the only mixed chemical ordering occurred in the only case studied where both coalescing NPs melted, allowing for their atoms to form an amorphous random solution. Simulations run at a temperature between the *T*_melt_'s of two NPs, exploited this difference in *T*_melt_, as discussed above, but, for practical reasons, involved different-size NPs; it would be interesting to confirm their results with equal-size NPs, although these would involve larger NPs and elevated temperatures. Further, it is a formidable but interesting challenge for the future to reproduce specific simulated configurations experimentally utilising gas-phase synthesis, since various current experimental setups around the world facilitate good control of the growth conditions (*e.g.*, ref. 61).

## Conflicts of interest

The authors declare no conflict of interest.

## Note added after first publication

This article replaces the version published on the 20th of October, which contained errors in eqn (1) and eqn (5).

## Supplementary Material
